# The correlation between the systemic inflammation response index and 6-month readmission risk in patients with hypertensive heart disease-related heart failure and its predictive model

**DOI:** 10.3389/fmed.2026.1799853

**Published:** 2026-05-28

**Authors:** Suqin Wang, Hongzhi Liu, Yuxiao Sun, Fang Yuan

**Affiliations:** 1 Heart Failure Ward of Fuwai Central China Cardiovascular Hospital, Henan Provincial People’s Hospital Heart Center, Zhengzhou, Henan, China; 2 Coronary Heart Disease Unit 4 of Fuwai Central China Cardiovascular Hospital, Henan Provincial People’s Hospital Heart Center, Zhengzhou, Henan, China

**Keywords:** heart failure, hypertensive heart disease, nomogram, predictive model, readmission, SIRI

## Abstract

**Objective:**

To investigate the association between the Systemic Inflammation Response Index (SIRI) and the risk of 6-month readmission in patients with hypertensive heart disease-related heart failure (HHD-HF), and develop and validate a nomogram prediction model integrating SIRI.

**Methods:**

A retrospective cohort of 158 hypertensive heart disease-related heart failure (HHD-HF) patients (June 2022–December 2024) was enrolled. Baseline clinical data, laboratory indicators, and cardiac structural and functional parameters were collected. Patients were split 7:3 into training (*n* = 111) and validation (*n* = 47) sets. LASSO regression was used for variable selection, and a multivariate logistic regression model incorporating SIRI was constructed and visualized as a nomogram. The model’s discriminative ability, calibration, and clinical utility were evaluated using receiver operating characteristic (ROC) curves, calibration curves, and decision curve analysis (DCA). Additionally, SHapley Additive exPlanations (SHAP) analysis was employed to interpret the contribution and directionality of SIRI and other predictors at both global and individual levels. Spearman rank correlation was used to explore the associations between SIRI and cardiac structural parameters, renal function, and comorbidities.

**Results:**

The LVMI (OR = 1.02, *p* = 0.046), diabetes history (OR = 5.62, *p* = 0.028), and SIRI (OR = 2.88, *p* = 0.014) as independent predictors of 6-month readmission in HHD-HF patients. The nomogram showed discrimination in training and validation cohorts (AUC 0.950 and 0.948, respectively). SHAP analysis confirmed SIRI as the dominant contributor (mean |SHAP| ≈ 1.4). SIRI positively correlated with readmission risk (*R* = 0.610) and LVMI (*R* = 0.413), and negatively eGFR (*R* = −0.446, all *p* < 0.001). In the total cohort, SIRI alone yielded an AUC of 0.863 (cutoff 1.71). Compared with other markers in the training cohort, SIRI significantly outperformed CRP (*p* = 0.001), NLR (*p* < 0.001), and BNP (*p* < 0.001). Subgroup analysis by LVEF showed consistent performance of SIRI in HFrEF (AUC 0.868) and HFpEF (AUC 0.829, *P* for difference = 0.589), and SIRI remained an independent predictor in both phenotypes (HFrEF: OR = 3.65, *p* = 0.025; HFpEF: OR = 3.33, *p* = 0.038).

**Conclusion:**

This nomogram incorporating LVMI, diabetes history, and SIRI effectively predicts 6-month readmission in HHD-HF patients, with discriminative ability and good calibration.

## 1 Introduction

Heart Failure (HF) is the most common and severe end-stage manifestation of hypertensive heart disease. The Global Burden of Disease study indicates that approximately 55.5 million individuals worldwide were living with heart failure in 2021, with an age-standardized prevalence rate of 676.68 per 100,000 population. Hypertensive heart disease (HHD) stands as the second leading etiology, following ischemic heart disease, contributing to 22.53% of the heart failure disease burden (1, 2). This substantial patient population imposes a significant disease and economic burden on global public health systems. Despite advances in diagnostic and therapeutic strategies, HF patients continue to face the severe challenges of high mortality and readmission rates: a meta-analysis encompassing 1.5 million patients globally revealed that all-cause readmission rates for hospitalized HF patients are as high as 13.2% and 35.7% at 30 days and one year, respectively (3). Therefore, identifying effective prognostic assessment tools for early risk stratification and guiding personalized management in patients, particularly within the important subgroup of HHD, is of paramount clinical significance.

In recent years, chronic low-grade inflammation has been recognized as a core pathophysiological mechanism underlying the initiation and progression of hypertension, ultimately leading to myocardial remodeling and cardiac dysfunction (4). However, the clinical utility of traditional inflammatory biomarkers in risk prediction is limited. The acute-phase reactant C-reactive protein (CRP) is highly susceptible to transient fluctuations induced by infection or stress, rendering it unreliable for assessing the sustained inflammatory state in patients with stable chronic heart failure (5). Similarly, although the neutrophil-to-lymphocyte ratio (NLR) is widely used, it only reflects the relative proportion of two leukocyte subsets and fails to capture the complexity of immune dysregulation involved in monocyte-mediated tissue remodeling (6). In this context, the systemic inflammation response index (SIRI), a composite index integrating neutrophil, monocyte, and lymphocyte counts, provides a more comprehensive and reliable basis for evaluating systemic inflammatory status. By simultaneously monitoring innate immune activation (via neutrophils and monocytes) and adaptive immune suppression (via lymphocytes), this index more accurately reflects the state of inflammatory imbalance in the body compared to single biomarkers. It also offers the unique advantages of being easily accessible and highly cost-effective (7). Accumulating evidence suggests that SIRI holds significant predictive value for the prognosis of various cardiovascular diseases, including heart failure. For instance, in patients with chronic HF, elevated SIRI levels are independently associated with a significantly increased risk of all-cause mortality (8). However, the predictive value of SIRI in the HHD-HF population, particularly for assessing short-term readmission risk, remains inadequately investigated. Furthermore, the pathophysiological links between SIRI and cardiac structural remodeling, renal function, and metabolic comorbidities within this subgroup have not been systematically elucidated. Additionally, existing predictive models for heart failure (HF) readmission primarily rely on traditional regression methods. While these methods can identify risk factors, they offer limited transparency regarding how each variable influences the prediction for a specific patient.

Therefore, this study aims to systematically investigate the correlation between SIRI and the risk of readmission within 6 months after discharge in HHD-HF patients, and to construct a predictive model combining SIRI with conventional clinical indicators using a visualized nomogram. This approach is intended to provide a new practical tool for early risk stratification and clinical decision-making in this specific population.

## 2 Method

### 2.1 Participants

A retrospective cohort study design was adopted. The study subjects were patients with HHD-HF admitted to the Heart Failure Ward of Fuwai Central China Cardiovascular Hospital between June 2022 and December 2024. The diagnostic criteria referred to the “*Chinese Guidelines for the Prevention and Treatment of Hypertension (2018 Revision)*” (9) and the “*Chinese Guidelines for the Diagnosis and Treatment of Heart Failure 2018*” (10). A total of 158 patients meeting the criteria were included in this study. Based on whether patients were rehospitalized for heart failure within six months after discharge, the training set was divided into a readmission group (*n* = 66) and a non-readmission group (*n* = 45). The study was approved by the Medical Research Ethics Review Committee of Heart Failure Ward of Fuwai Central China Cardiovascular Hospital (Ethics Approval No.: 2024–81). Informed consent was waived in accordance with the committee’s guidelines for retrospective research.

*Inclusion criteria*: Patients meeting all of the following criteria were included in this study: (1) Diagnosed with hypertensive heart disease accompanied by heart failure; (2) Age ≥ 18 years; (3) Possess complete baseline clinical data; (4) Provided signed informed consent.

*Exclusion criteria*: (1) Secondary hypertension or heart failure primarily attributed to other etiologies (such as severe valvular heart disease, congenital heart disease, acute coronary syndrome occurring within the past 3 months, myocarditis, pericardial disease, or primary cardiomyopathy); (2) Presence of severe non-cardiac diseases with an expected lifespan of less than 6 months, including active malignancy, severe hepatic insufficiency (Child-Pugh Class C), end-stage renal disease requiring renal replacement therapy, or severe systemic infectious diseases; (3) History of cardiac surgery or percutaneous coronary intervention within the past 3 months; (4) Pregnancy or lactation; (5) Incomplete follow-up data or loss to follow-up during the 6-month observation period.

*Follow-up*: The readmission status at 6 months was confirmed through dual verification: (1) structured telephone follow-up conducted by trained research nurses at 1, 3, and 6 months post-discharge, and (2) review of electronic medical record (EMR) systems at our hospital and four major collaborating hospitals in the Zhengzhou region to capture readmissions to other facilities. No patients were lost to follow-up during the 6-month observation period.

### 2.2 Sample size

This study was a retrospective predictive modeling study designed to develop and validate a model for predicting the 6-month readmission risk in patients with HHD-HF. Sample size estimation followed commonly accepted empirical guidelines for predictive model development. First, based on previous literature, the anticipated event rate for the primary outcome (heart failure-related readmission within 6 months) was estimated to be approximately 20–30%(3). Second, in predictive modeling, to avoid overfitting and ensure model stability, it is generally recommended to have at least 5 to 10 outcome events per candidate predictor variable initially considered. Although a larger set of candidate predictors was included for analysis and LASSO regression was planned for variable selection, we conservatively estimated that approximately 8 core variables might be retained after dimensionality reduction. To maintain sufficient modeling degrees of freedom, the calculation was based on this number. Therefore, the minimum required number of outcome events was calculated as 8 (variables) × 5 (events per variable) = 40 events. Given the 30% expected event rate, the theoretical minimum total sample size was approximately 40/0.30 ≈ 134 patients. Furthermore, considering potential issues with data completeness or missing information inherent in retrospective clinical studies, a final cohort of 158 eligible patients was included. This ensured the sample size was adequate for both model construction and subsequent internal validation.

### 2.3 Clinical data collection

Data on the following variables were collected from patients in both groups: sex, age, body mass index (BMI), smoking history, alcohol drinking history, history of diabetes mellitus (DM), history of coronary heart disease (CHD), creatinine (Cr), estimated glomerular filtration rate (eGFR), B-type natriuretic peptide (BNP), low-density lipoprotein cholesterol (LDL-C), CRP, SIRI, NLR, platelet-to-lymphocyte ratio (PLR), monocyte-to-HDL cholesterol ratio (MHR), neutrophil-to-HDL cholesterol ratio (NHR), left ventricular posterior wall thickness (LVPW), relative wall thickness (RWT), left ventricular end-diastolic dimension (LVEDD), left atrial dimension (LAD), left ventricular mass index (LVMI), left ventricular ejection fraction (LVEF), early mitral inflow velocity to mitral annular early diastolic velocity ratio (E/e’), tricuspid regurgitation velocity (TRV), pulmonary artery systolic pressure (PASP), and types of cardiac remodeling [concentric hypertrophy (CH), concentric re modeling (CR), eccentric hypertrophy (EH), normal geometry (NG)]. SIRI = Neutrophil × Monocyte/Lymphocyte; NLR = Neutrophil/Lymphocyte; PLR = Platelet/Lymphocyte; MHR = Monocyte/HDL-C; NHR = Neutrophil/HDL-C. All laboratory parameters, including complete blood count and inflammatory markers, were measured using blood samples collected within 24 h of hospital admission.

Left ventricular geometry was classified according to the Ganau method into four categories based on LVMI and RWT (11, 12): NG (LVMI ≤ 115 g/m^2^ for men/95 g/m^2^ for women and RWT ≤ 0.42), CR (LVMI ≤ 115 g/m^2^ for men/95 g/m^2^ for women and RWT > 0.42), EH (LVMI > 115 g/m^2^ for men/95 g/m^2^ for women and RWT ≤ 0.42), and CH (LVMI > 115 g/m^2^ for men/95 g/m^2^ for women and RWT > 0.42). LVMI was calculated using the Devereux formula and indexed to body surface area, while RWT was defined as (interventricular septal thickness + left ventricular posterior wall thickness)/left ventricular end-diastolic diameter.

### 2.4 Statistical analysis

All statistical analyses were performed using R software (version 4.2.2) and MSTATA software.^1^ Data were randomly split into a training set and a validation set in a 7:3 ratio, and variables were compared between them. Continuous variables following a normal distribution are presented as mean ± standard deviation, while non-normally distributed data are presented as median (interquartile range). In univariate analysis, categorical variables were analyzed using the chi-square test or Fisher’s exact test, and continuous variables were analyzed using the Student’s *t*-test or the rank-sum test. Before variable selection, multicollinearity among the candidate predictors was assessed using the variance inflation factor (VIF). A VIF > 10 was considered indicative of significant multicollinearity, and such variables would be considered for exclusion or combination. In the training set, least absolute shrinkage and selection operator (LASSO) logistic regression analysis was employed for multivariate analysis to screen for independent risk factors and construct a predictive nomogram. LASSO regression was performed using 10-fold cross-validation, and the optimal penalty parameter *λ* was selected based on the “1-standard error” criterion. Model performance was evaluated using the receiver operating characteristic (ROC) curve and calibration curve. The area under the ROC curve (AUC) ranges from 0.5 (no discriminative ability) to 1 (perfect discrimination). To further assess calibration, the Hosmer-Lemeshow (H-L) goodness-of-fit test was applied, with *p* > 0.05 indicating adequate calibration. Additionally, 1,000 cycles of Bootstrap resampling were performed to calculate the optimism-corrected C-index and calibration slope. Decision curve analysis (DCA) was also conducted to determine the net benefit threshold of the prediction. To enhance the transparency and clinical interpretability of the nomogram, this study employed the SHapley Additive exPlanations (SHAP) method for *post hoc* interpretation of the optimal model. SHAP values were calculated using the ‘shapviz’ package in R, based on the final multivariate logistic regression model. For each prediction, 1,000 Monte Carlo samples were used to approximate SHAP values. The baseline expected value E[f(x)] was defined as the mean predicted probability of readmission in the training cohort. The global and individual SHAP values were calculated to quantify the contribution and direction of each feature to model predictions. A bar plot of mean absolute SHAP values was generated to evaluate the overall importance of features; a beeswarm plot was used to visualize the distribution between feature values and SHAP values; and waterfall plots were drawn for representative individual cases to illustrate the mechanism of individual predictions. Interpretation of SHAP values: positive values indicate that the feature shifts the prediction toward high-risk/positive outcomes, while negative values indicate a shift toward low-risk/negative outcomes. To explore the relationship between SIRI and clinical indicators, Spearman’s rank-order correlation was used to assess monotonic associations between variables. A *p*-value < 0.05 was considered statistically significant.

## 3 Results

### 3.1 Comparison of patient characteristics between the training set and the validation set

To evaluate the balance between groups after data splitting, this study randomly divided 158 HHD-HF patients into a training set (*n* = 111) and an internal validation set (*n* = 47) in a 7:3 ratio, and compared all baseline variables. As shown in Table 1, there were no statistically significant differences between the two groups in most demographic characteristics, clinical history, laboratory tests, cardiac structure and function parameters, or inflammatory markers (*p* > 0.05), indicating that the baseline data were comparable and balanced. Only a few variables differed between the groups: the median levels of PASP, NLR, SIRI, and NHR in the validation set were lower than those in the training set (*p* < 0.05). Nevertheless, these levels remained within clinically common ranges in both groups, with minimal absolute differences between groups, which did not affect the validation of the model constructed in the training set (Table 1).

**Table 1:** Comparison of baseline patient characteristics between training set and validation set.

Characteristic	Training cohort *N* = 111	Internal test cohort *N* = 47	*χ^2^*/*Z*/*t*	*p*-value
Gender, *n* (%)			0.096	0.757^1^
Female	31 (27.93%)	12 (25.53%)		
Male	80 (72.07%)	35 (74.47%)		
Age (year, Mean ± SD)	53.72 ± 17.24	53.93 ± 16.22	−0.072	0.943^2^
LVPW [mm, *M* (*Q_1_*, *Q_3_*)]	10.00 (9.00, 12.00)	10.00 (9.00, 13.00)	−0.541	0.590^3^
RWT [*M* (*Q_1_*, *Q_3_*)]	0.38 (0.32, 0.45)	0.37 (0.32, 0.42)	−1.286	0.199^3^
LVEF [%, *M* (*Q_1_*, *Q_3_*)]	34.64 (29.40, 54.60)	39.90 (31.50, 48.00)	−0.578	0.564^3^
E/e’ [*M* (*Q_1_*, *Q_3_*)]	17.00 (14.20, 22.00)	15.30 (12.00, 22.10)	−1.898	0.058^3^
TR [m/s, *M* (*Q_1_*, *Q_3_*)]	2.50 (2.10, 2.90)	2.30 (2.10, 2.70)	−1.329	0.184^3^
PASP [mmHg, *M* (*Q_1_*, *Q_3_*)]	37.50 (25.50, 45.90)	27.00 (22.50, 39.00)	−2.838	0.005^3^
LVEDD [mm, *M* (*Q_1_*, *Q_3_*)]	60.00 (50.00, 67.00)	59.00 (52.00, 65.00)	−0.392	0.696^3^
LAD [mm, *M* (*Q_1_*, *Q_3_*)]	45.00 (40.00, 50.00)	42.00 (40.00, 46.00)	−1.543	0.123^3^
LVMI [g/m^2^, *M* (*Q_1_*, *Q_3_*)]	146.52 (100.13, 200.38)	137.80 (103.72, 188.57)	−0.557	0.579^3^
Types of cardiac remodeling, *n* (%)			6.512	0.079^4^
CH	28 (25.23%)	14 (29.79%)		
CR	14 (12.61%)	1 (2.13%)		
EH	51 (45.95%)	19 (40.43%)		
NG	18 (16.22%)	13 (27.66%)		
BMI [kg/m^2^ *M* (*Q_1_*, *Q_3_*)]	29.1 (26.3, 32.7)	28.0 (25.5, 31.9)	−1.059	0.290^3^
Diabetes mellitus history, *n* (%)			0.978	0.323^1^
No	59 (53.15%)	29 (61.70%)		
Yes	52 (46.85%)	18 (38.30%)		
Coronary heart disease history, *n* (%)			2.680	0.102^1^
No	65 (58.56%)	34 (72.34%)		
Yes	46 (41.44%)	13 (27.66%)		
Smoking history, *n* (%)			0.009	0.927^1^
No	67 (60.36%)	28 (59.57%)		
Yes	44 (39.64%)	19 (40.43%)		
Alcohol drinking history, *n* (%)			0.135	0.713^1^
No	65 (58.56%)	29 (61.70%)		
Yes	46 (41.44%)	18 (38.30%)		
Cr [μmol/L, *M* (*Q_1_*, *Q_3_*)]	84.00 (70.00, 106.00)	77.00 (64.00, 98.00)	−1.415	0.158^3^
eGFR [*M* (*Q_1_*, *Q_3_*)]	65.59 (51.08, 83.67)	68.65 (55.00, 95.38)	−1.364	0.173^3^
BNP [pg/mL, *M* (*Q_1_*, *Q_3_*)]	1615.00 (570.86, 3551.49)	1030.00 (309.74, 2277.00)	−1.550	0.122^3^
NLR [*M* (*Q_1_*, *Q_3_*)]	2.68 (1.68, 4.09)	1.99 (1.44, 3.39)	−2.332	0.020^3^
SIRI [*M* (*Q_1_*, *Q_3_*)]	1.67 (1.01, 2.84)	1.12 (0.78, 2.35)	−2.033	0.042^3^
PLR [*M* (*Q_1_*, *Q_3_*)]	123.35 (96.91, 155.84)	127.56 (88.19, 154.92)	−0.034	0.974^3^
MHR [*M* (*Q_1_*, *Q_3_*)]	0.48 (0.33, 0.63)	0.44 (0.31, 0.64)	−0.430	0.669^3^
NHR [*M* (*Q_1_*, *Q_3_*)]	4.87 (3.74, 6.38)	4.19 (2.70, 5.84)	−2.352	0.019^3^
LDL-c [mmol/L, *M* (*Q_1_*, *Q_3_*)]	2.22 (1.72, 2.84)	2.20 (1.65, 2.96)	−0.118	0.908^3^
CRP[mg/L, *M* (*Q_1_*, *Q_3_*)]	4.06 (1.99, 10.04)	3.05 (1.22, 11.23)	−0.919	0.359^3^

1 Pearson’s Chi-squared test.

2 Welch Two Sample *t*-test.

3 Wilcoxon rank sum test.

4 Fisher’s exact test.

Patients in the training set were divided into a non-readmission group (*n* = 66) and a readmission group (*n* = 45) based on whether they were readmitted for heart failure within 6 months after discharge. Univariate analysis revealed significant differences between the readmission group and the non-readmission group in multiple key baseline characteristics (Table 2). In terms of cardiac structure and function, the readmission group exhibited poorer conditions, including higher RWT, LVMI, E/e’ ratio, and PASP, as well as lower LVEF and eGFR (all comparisons *p* < 0.01). Additionally, systemic inflammation levels were significantly elevated in the readmission group, with median values of the SIRI and NLR markedly higher than those in the non-readmission group (all *p* < 0.001). Furthermore, the readmission group had a heavier burden of comorbidities, with significantly higher proportions of patients with a history of diabetes and coronary heart disease (both *p* < 0.001), as well as significantly elevated levels of heart failure biomarkers BNP and CRP (*p* < 0.001). However, no statistically significant differences were observed between the two groups in terms of gender, age, body mass index, smoking and alcohol consumption history, lipid levels, or certain cardiac chamber diameter measurements.

**Table 2:** Baseline characteristics of patients in the readmission group and non-readmission group in the training and validation sets.

Characteristic	Cohort (training cohort), *N* = 111				Cohort (internal test cohort), *N* = 47			
	Non-readmission group *N* = 66	Readmission group *N* = 45	*χ^2^*/*Z*/*t*	*p*-value^1^	Non-readmission group *N* = 32	Readmission group *N* = 15	*χ^2^*/*Z*/*t*	*p*-value^2^
Gender, *n* (%)			0.060	0.807			0.014	>0.999
Female	19 (28.79%)	12 (26.67%)			8 (25.00%)	4 (26.67%)		
Male	47 (71.21%)	33 (73.33%)			24 (75.00%)	11 (73.33%)		
Age (year, Mean ± SD)	51.61 ± 16.60	56.81 ± 17.87	−1.547	0.125	52.37 ± 16.80	57.25 ± 14.88	−1.004	0.323
LVPW [mm, *M* (*Q_1_*, *Q_3_*)]	10.00 (9.00, 12.00)	11.00 (10.00, 12.50)	−1.148	0.252	10.00 (9.00, 12.50)	11.00 (8.00, 13.00)	−0.115	0.917
RWT [*M* (*Q_1_*, *Q_3_*)]	0.37 (0.31, 0.40)	0.45 (0.37, 0.55)	−4.536	<0.001	0.36 (0.32, 0.40)	0.39 (0.31, 0.53)	−1.267	0.209
LVEF [%, *M* (*Q_1_*, *Q_3_*)]	42.52 (32.41, 59.89)	31.50 (23.63, 39.00)	−4.349	<0.001	43.57 (32.03, 57.56)	31.50 (29.00, 36.00)	−3.140	0.002
E/e’ [*M* (*Q_1_*, *Q_3_*)]	15.30 (13.00, 19.23)	20.55 (16.20, 25.10)	−4.683	<0.001	13.60 (11.05, 16.83)	21.50 (15.40, 25.50)	−3.436	<0.001
TR [m/s, *M* (*Q_1_*, *Q_3_*)]	2.55 (2.20, 3.00)	2.36 (2.10, 2.60)	−1.909	0.057	2.26 (2.12, 2.67)	2.40 (2.00, 2.70)	−0.161	0.881
PASP [mmHg, *M* (*Q_1_*, *Q_3_*)]	33.60 (23.18, 41.00)	40.50 (30.75, 51.00)	−2.760	0.006	26.05 (20.70, 28.95)	34.50 (28.50, 43.00)	−3.061	0.002
LVEDD [mm, *M* (*Q_1_*, *Q_3_*)]	58.00 (48.00, 66.00)	61.00 (53.00, 69.00)	−1.620	0.106	56.00 (51.25, 64.75)	60.00 (54.00, 66.00)	−1.211	0.230
LAD [mm, *M* (*Q_1_*, *Q_3_*)]	45.00 (40.00, 49.00)	44.00 (40.50, 50.50)	−0.394	0.696	42.00 (40.00, 45.75)	45.00 (40.00, 49.00)	−0.847	0.403
LVMI [g/m^2^, *M* (*Q_1_*, *Q_3_*)]	121.60 (87.62, 163.49)	201.82 (152.98, 244.79)	−5.736	<0.001	117.70 (89.70, 154.28)	188.57 (143.05, 234.43)	−3.776	<0.001
Types of cardiac remodeling, *n* (%)			4.049	0.256			3.105	0.396
CH	17 (25.76%)	11 (24.44%)			9 (28.13%)	5 (33.33%)		
CR	11 (16.67%)	3 (6.67%)			1 (3.13%)	0 (0.00%)		
EH	26 (39.39%)	25 (55.56%)			11 (34.38%)	8 (53.33%)		
NG	12 (18.18%)	6 (13.33%)			11 (34.38%)	2 (13.33%)		
BMI [kg/m^2^ *M* (*Q_1_*, *Q_3_*)]	28.40 (26.30, 31.80)	30.1 (26.8, 33.4)	−1.313	0.190	28.30 (26.70, 31.70)	26.63 (24.30, 32.00)	−1.175	0.244
Diabetes mellitus history, *n* (%)			29.078	<0.001			7.503	0.006
No	49 (74.24%)	10 (22.22%)			24 (75.00%)	5 (33.33%)		
Yes	17 (25.76%)	35 (77.78%)			8 (25.00%)	10 (66.67%)		
Coronary heart disease history, *n* (%)			23.495	<0.001			3.978	0.079
No	51 (77.27%)	14 (31.11%)			26 (81.25%)	8 (53.33%)		
Yes	15 (22.73%)	31 (68.89%)			6 (18.75%)	7 (46.67%)		
Smoking history, *n* (%)			1.562	0.211			1.524	0.217
No	43 (65.15%)	24 (53.33%)			21 (65.63%)	7 (46.67%)		
Yes	23 (34.85%)	21 (46.67%)			11 (34.38%)	8 (53.33%)		
Alcohol drinking history, *n* (%)			0.851	0.356			0.027	0.869
No	41 (62.12%)	24 (53.33%)			20 (62.50%)	9 (60.00%)		
Yes	25 (37.88%)	21 (46.67%)			12 (37.50%)	6 (40.00%)		
Cr [μmol/L, *M* (*Q_1_*, *Q_3_*)]	85.00 (70.00, 105.25)	83.00 (73.00, 109.00)	−0.099	0.923	75.50 (63.00, 91.25)	78 (75, 113)	−1.222	0.226
eGFR [*M* (*Q_1_*, *Q_3_*)]	76.03 (62.34, 92.80)	53.97 (43.40, 62.83)	−5.820	<0.001	80.47 (63.33, 98.80)	54 (42, 66)	−3.560	<0.001
BNP [pg/mL, *M* (*Q_1_*, *Q_3_*)]	1179.50 (393.06, 2057.95)	2216.16 (1362.27, 5115.50)	−3.448	<0.001	633.40 (184.87, 2038.41)	1833.00 (572.80, 7963.00)	−2.533	0.011
NLR [*M* (*Q_1_*, *Q_3_*)]	2.07 (1.50, 2.82)	3.71 (2.84, 6.73)	−5.664	<0.001	1.80 (1.33, 2.46)	3.05 (1.69, 4.68)	−2.373	0.017
SIRI [*M* (*Q_1_*, *Q_3_*)]	1.11 (0.84, 1.67)	2.72 (2.00, 4.87)	−6.847	<0.001	1.00 (0.70, 1.52)	2.53 (1.24, 3.93)	−3.264	<0.001
PLR [*M* (*Q_1_*, *Q_3_*)]	120.80 (93.60, 155.53)	125.60 (98.38, 157.77)	−0.646	0.520	131.65 (102.44, 162.36)	100 (75, 148)	−1.666	0.098
MHR [*M* (*Q_1_*, *Q_3_*)]	0.45 (0.32, 0.63)	0.51 (0.34, 0.64)	−0.796	0.428	0.45 (0.30, 0.64)	0.43 (0.33, 0.76)	−0.457	0.656
NHR [*M* (*Q_1_*, *Q_3_*)]	4.53 (3.51, 6.37)	5.03 (3.95, 6.72)	−0.679	0.499	4.23 (2.50, 5.81)	3.88 (2.97, 8.12)	−0.434	0.676
LDL-c [mmol/L, *M* (*Q_1_*, *Q_3_*)]	2.17 (1.70, 3.08)	2.25 (1.67, 2.73)	−0.045	0.966	2.29 (1.68, 2.97)	2.10 (1.45, 2.59)	−0.571	0.576
CRP[mg/L, *M* (*Q_1_*, *Q_3_*)]	2.84 (1.43, 6.85)	7.39 (3.73, 15.90)	−3.742	<0.001	2.62 (1.19, 9.06)	8.69 (1.26, 14.74)	−1.255	0.216

1 Pearson’s Chi-squared test; Welch Two Sample *t*-test; Wilcoxon rank sum test.

2 Fisher’s exact test; Welch Two Sample *t*-test; Wilcoxon rank sum test; Wilcoxon rank sum exact test; Pearson’s Chi-squared test.

### 3.2 The results of the correlation analysis between SIRI and clinical indicators

**Figure 1:**
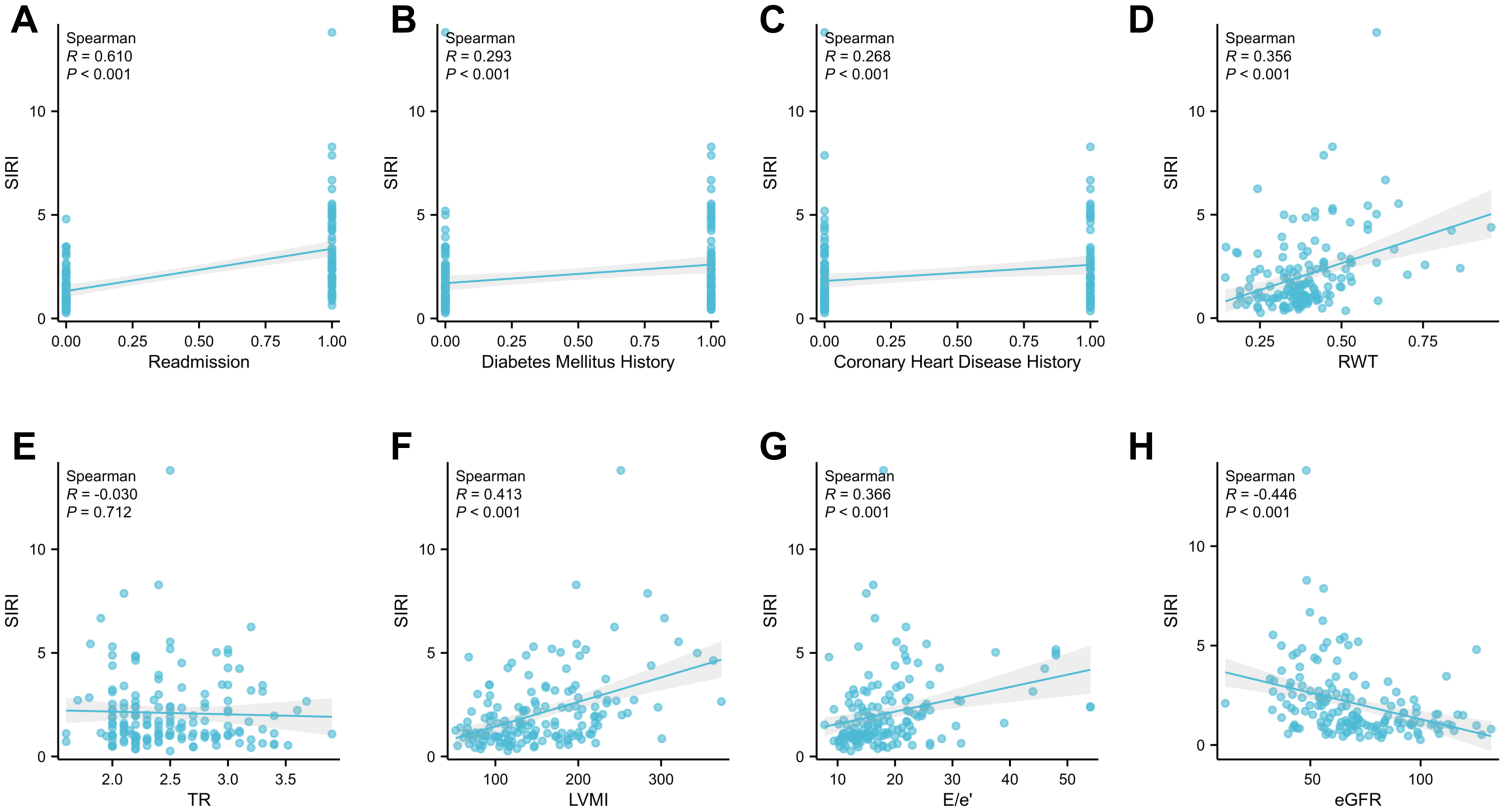
Spearman rank correlation analysis of the correlation between SIRI and clinical variables. The scatter plots and Spearman correlation coefficients (R) between the SIRI and the following variables are shown: **(A)** Readmission; **(B)** Diabetes Mellitus History; **(C)** Coronary Heart Disease History; **(D)** RWT; **(E)** TR; **(F)** LVMI; **(G)** E/e'; **(H)** eGFR. The shaded areas represent the 95% confidence intervals of the fitted lines. For binary variables **(A–C)**, 0 and 1 represent the absence/presence of the outcome or comorbidity, respectively. A *P*-value <0.05 was considered statistically significant.

Spearman rank correlation analysis was used to explore the associations between SIRI and clinical outcomes, comorbidities, as well as cardiac and renal function indicators (Figure 1). As shown in Figure 1A, SIRI was significantly positively correlated with the risk of readmission (*R* = 0.610, *p* < 0.001), demonstrating the strongest association among all tested variables. In terms of comorbidities, SIRI showed weak to moderate positive correlations with a history of diabetes (Figure 1B: *R* = 0.293, *p* < 0.001) and a history of coronary heart disease (Figure 1C: *R* = 0.268, *p* < 0.001). Analysis of cardiac structure and function indicators (Figures 1D,F,G) revealed that SIRI was significantly positively correlated with RWT (*R* = 0.356), LVMI (*R* = 0.413), and E/e’ (*R* = 0.366) (all *p* < 0.001). Additionally, SIRI showed a moderate negative correlation with eGFR (Figure 1E: *R* = −0.446, *p* < 0.001), while no significant correlation was observed with TR severity (Figure 1H: *R* = −0.030, *p* = 0.712).

### 3.3 Variable selection and VIF analysis

**Figure 2:**
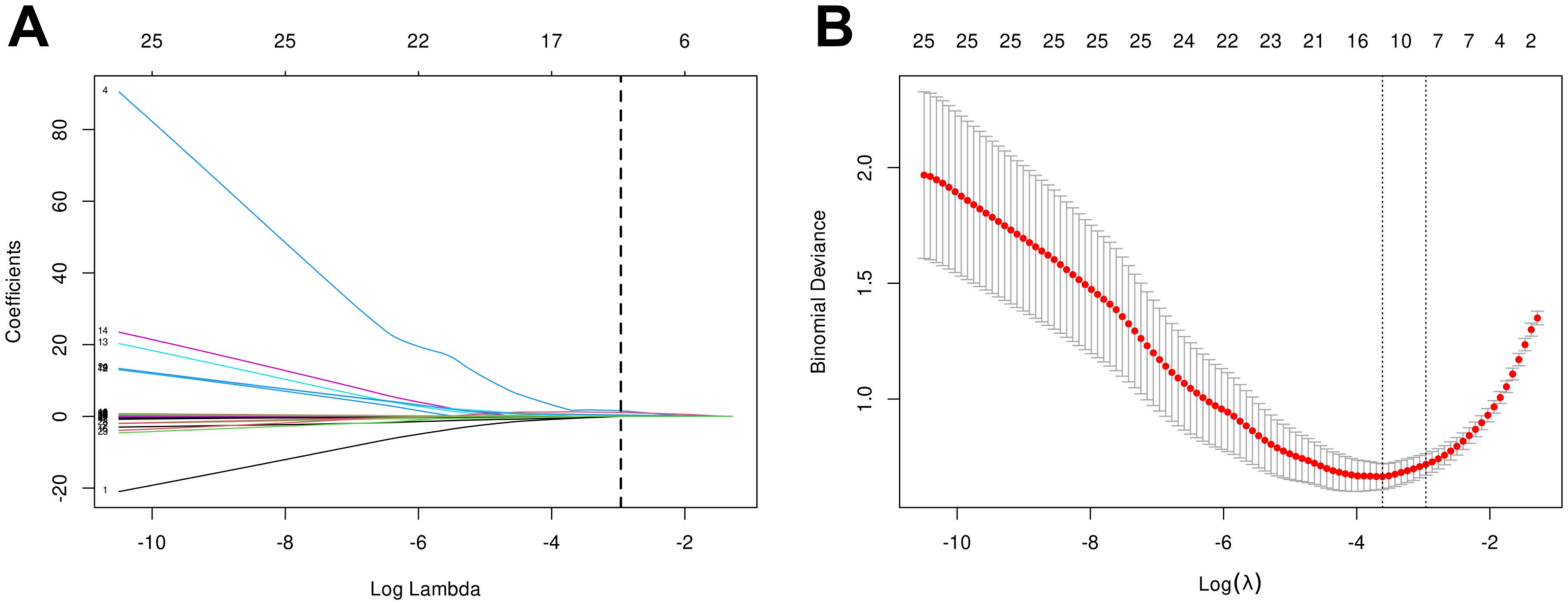
**(A)** Plot of the LASSO regression coefficient profiles (λ = 0.0517572302215329). Each colored line represents the trajectory of independent variable coefficients as the penalty parameter λ varies. The *x*-axis displays log(λ) (indicating penalty strength), while the *y*-axis shows the absolute values of regression coefficients. When λ increases, coefficients approach zero, indicating variable elimination; when λ decreases, some coefficients increase and stabilize. **(B)** The penalty parameter λ is selected using ten-fold cross-validation, retaining 8 variables.

All candidate predictors were incorporated into the model. Subsequently, LASSO regression analysis was performed on the training cohort to reduce these predictors to 8 latent variables. The coefficient values are listed in the Table 3, with the coefficient distribution diagram shown in the Figure 2. The figure also displays the cross-validation error curve of the LASSO regression model. After cross-validation, the most regularized and simplified model with error values within one standard error range of the minimum was ultimately selected, containing 8 variables.

**Table 3:** The coefficients of LASSO regression analysis.

Variable	Coefficient
(Intercept)	−2.622475785
RWT	1.544138079
E/e’	0.007832634
TR (m/s)	−0.023364410
LVMI (g/m^2^)	0.007584906
Diabetes Mellitus History-1	1.069579049
Coronary Heart Disease History-1	0.356173006
eGFR (mL/min/1.73 m^2^)	−0.018249680
SIRI	0.388571601

**Figure 3:**
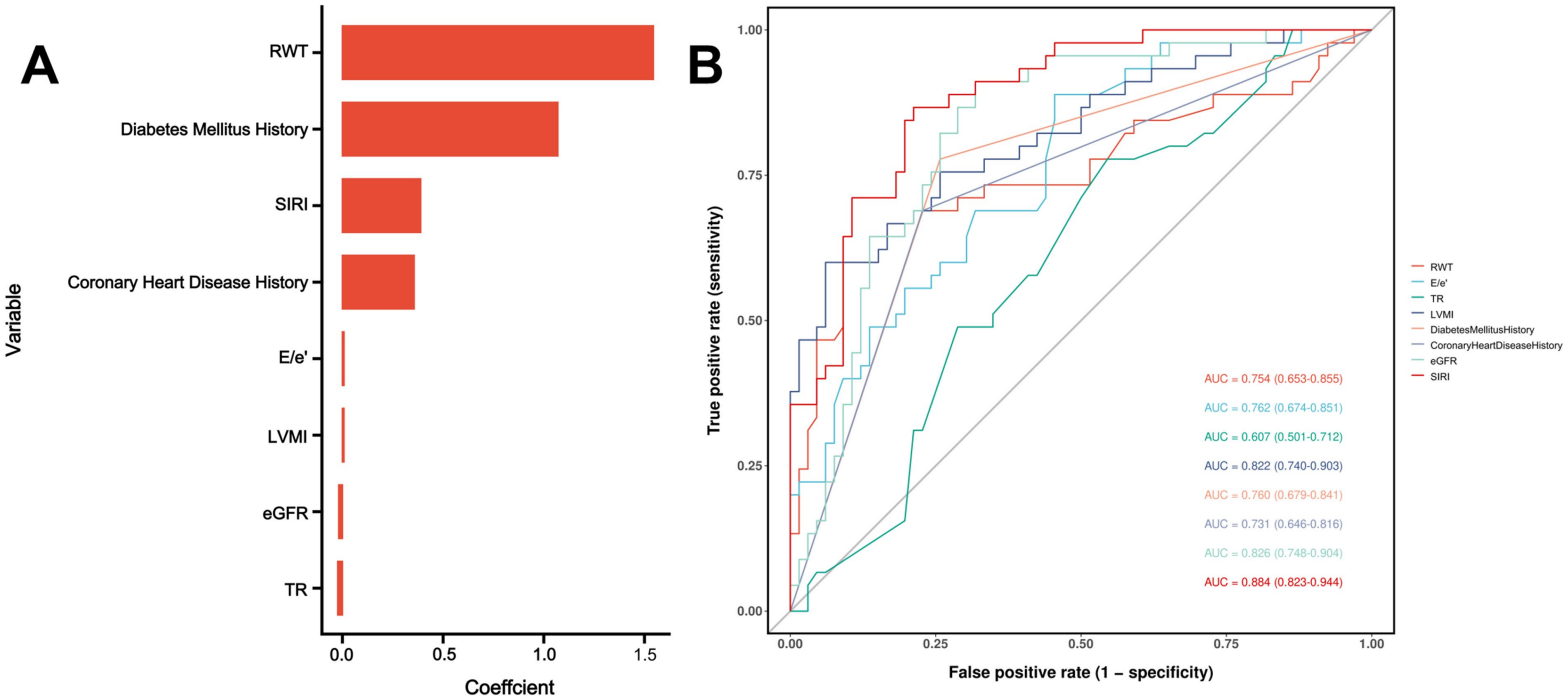
**(A)** LASSO-selected predictors and corresponding coefficients. Only variables with non-zero coefficients after LASSO selection are shown. **(B)** Comparison of ROC Curves for Individual Predictive Variables. ROC curves for individual predictors assessed in univariate logistic regression.

As shown in Figure 3, the ROC analysis of the abovementioned variables yielded AUC values greater than 0.5. The ROC curve analysis demonstrated the discriminative ability of individual predictors for the readmission group. The AUC for LVMI was 0.822 (95% CI: 0.740–0.903), closely followed by GFR with an AUC of 0.826 (95% CI: 0.748–0.904), and the SIRI showed the highest predictive value with an AUC of 0.884 (95% CI = 0.823–0.944). Moderately high predictive accuracies were observed for E/e’ (AUC = 0.762; 95% CI: 0.674–0.851), diabetes mellitus history (AUC = 0.760; 95% CI: 0.679–0.841), RWT (AUC = 0.754; 95% CI: 0.653–0.855), and coronary heart disease history (AUC = 0.731; 95% CI: 0.646–0.816). In contrast, the predictive performance of TR was comparatively lower, with an AUC of 0.607 (95% CI: 0.501–0.712).

VIF analysis was performed for all candidate predictors selected by LASSO. The results showed that all VIF values were below 2.0, with LVMI (VIF = 1.504) and SIRI (VIF = 1.437) demonstrating no significant multicollinearity (Supplementary Table 1).

### 3.4 Multivariate logistic regression analysis

The selected predictors were incorporated into the logistic regression model. The model formula is as follows: Risk of 6-month readmission = 1 / (1 + exp.(− [−4.398 + 4.263 × RWT (dimensionless) + 0.083 × E/e′ (dimensionless) - 1.262 × TR (m/s) + 0.015 × LVMI (g/m^2^) + 1.727 × Diabetes Mellitus History (1 = Yes) + 0.599 × Coronary Heart Disease History (1 = Yes) - 0.024 × eGFR (mL/min/1.73 m^2^) + 1.058 × SIRI (dimensionless)])). Table 4 presents the logistic regression analysis results of the influencing factors for 6-month readmission in HHD-HF patients.

**Table 4:** Results of multivariate logistic regression for training cohort.

Variables	*β*	S. E	Z	*P*	OR (95%CI)
Intercept	−4.398	3.475	−1.266	0.206	0.012 (0.000–11.170)
Diabetes mellitus history					
No					1.000 (Reference)
Yes	1.727	0.787	2.193	0.028	5.621 (1.202–26.293)
Coronary heart disease history					
No					1.000 (Reference)
Yes	0.599	0.804	0.745	0.457	1.820 (0.376–8.806)
RWT	4.263	3.958	1.077	0.281	71.054 (0.030–166235.593)
E/e’	0.083	0.078	1.055	0.291	1.086 (0.932–1.267)
TR (m/s)	−1.262	0.919	−1.373	0.170	0.283 (0.047–1.715)
LVMI (g/m^2^)	0.015	0.008	1.994	0.046	1.015 (1.001–1.031)
SIRI	1.058	0.429	2.466	0.014	2.882 (1.242–6.684)
eGFR (mL/min/1.73 m^2^)	−0.024	0.019	−1.280	0.200	0.977 (0.942–1.013)

CI, confidence interval; OR, odds ratio.

In the multivariate logistic regression analysis of the training cohort, LVMI (OR 1.015, 95% CI 1.001–1.031, *p* = 0.046), history of diabetes mellitus (with the absence of diabetes as the reference; OR 5.621, 95% CI 1.202–26.293, *p* = 0.028 for the presence of diabetes), and SIRI (OR 2.882, 95% CI 1.242–6.684, *p* = 0.014) were identified as statistically significant independent predictors. Higher LVMI values, the presence of diabetes, and elevated SIRI were associated with increased odds of the outcome event. In contrast, RWT (OR 71.054, 95% CI 0.030–166235.593, *p* = 0.281), E/e’ (OR 1.086, 95% CI 0.932–1.267, *p* = 0.291), TR (OR 0.283, 95% CI 0.047–1.715, *p* = 0.170), history of coronary heart disease (with no history as reference; OR 1.820, 95% CI 0.376–8.806, *p* = 0.457 for its presence), and eGFR (OR 0.977, 95% CI 0.942–1.013, *p* = 0.200) showed no statistically significant association with the outcome event (Table 4).

### 3.5 Evaluation and internal validation

**Figure 4:**
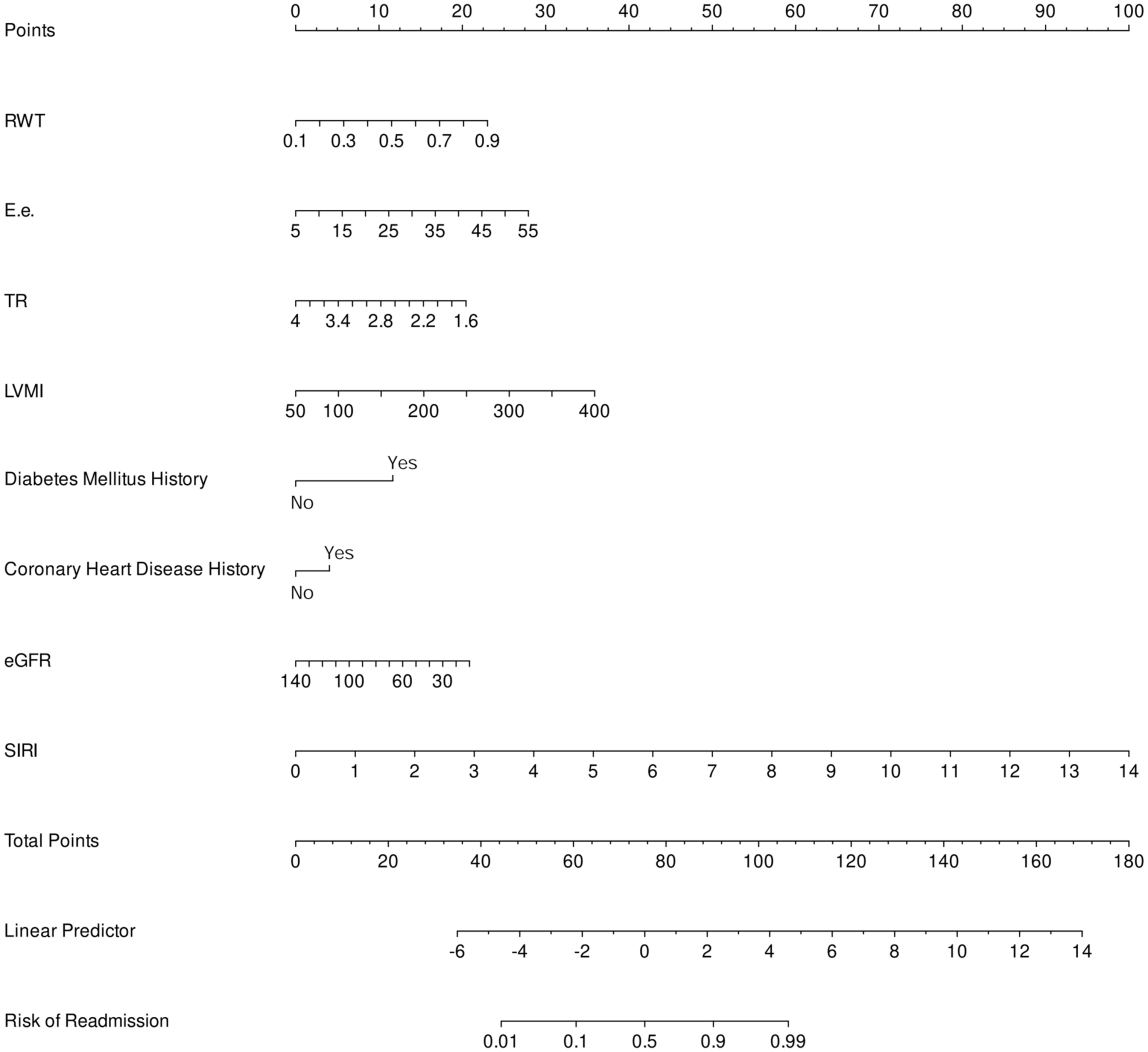
Nomogram for predicting the risk of readmission within six months in HHD-HF patients.

The constructed prediction model is presented in the form of a risk nomogram. This nomogram projects the original measured or categorical outcomes of the eight independent variables in the model onto the top point scale line to obtain corresponding points. These points are then summed to yield a total score, which is subsequently projected downward onto the bottom axis to predict the probability of six-month readmission risk for HHD-HF patients, as illustrated in Figure 4.

**Figure 5:**
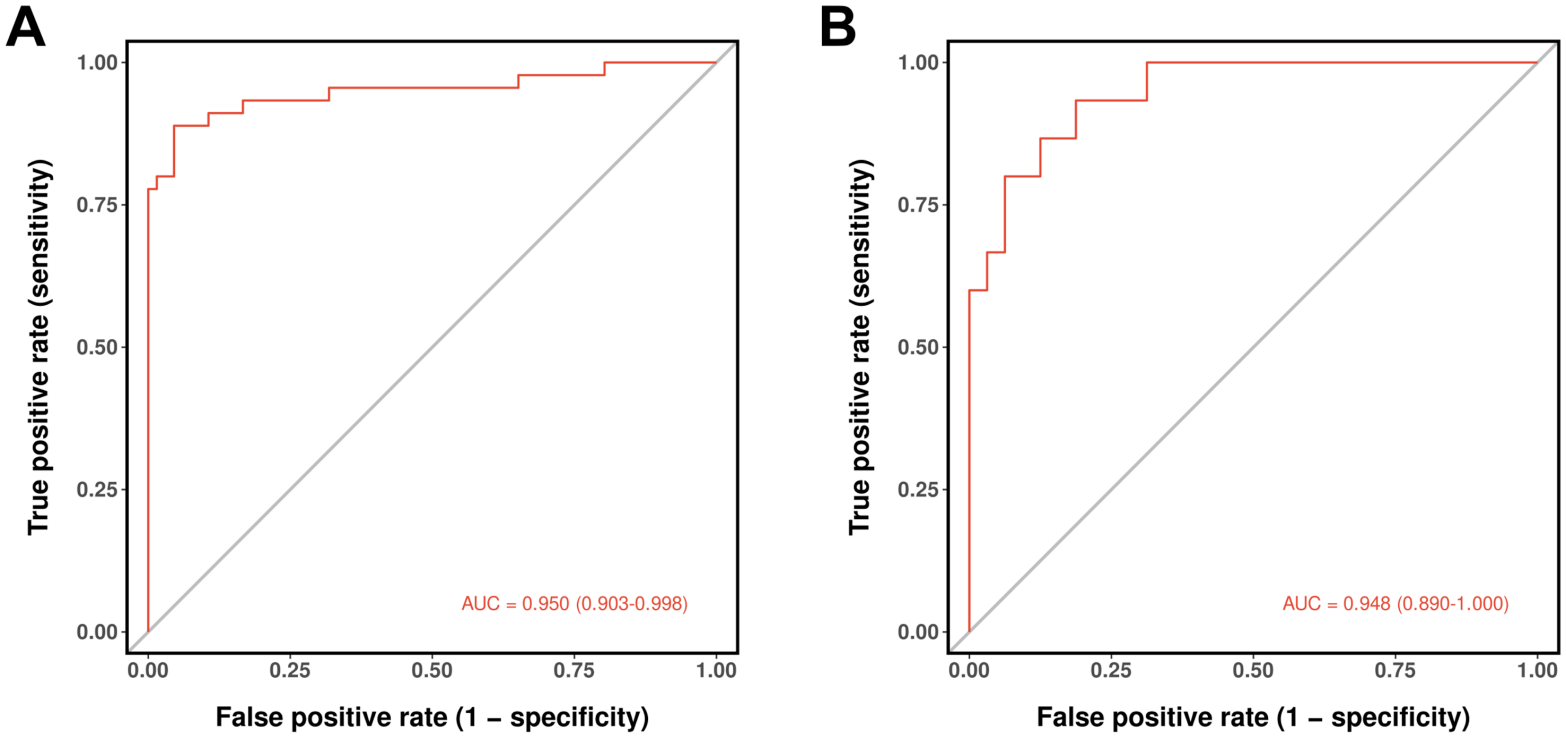
ROC curve of the readmission prediction model for HHD-HF patients. **(A)** Training set. **(B)** Validation set.

The AUCs of the model in the different cohorts were shown in the Figure 5. The ROC curve analysis for the predictive model demonstrated excellent discriminatory performance across both cohorts. In the training cohort, the AUC was 0.950 (95% CI: 0.903–0.998). This high level of performance was maintained in the independent validation cohort, where the model achieved an AUC of 0.948 (95% CI: 0.890–1.000), indicating robust generalizability and a consistent ability to distinguish between the outcome groups.

To evaluate whether the significant baseline differences in PASP, NLR, SIRI, and NHR between the training and validation sets (Table 1) affected the generalizability of the nomogram, we stratified the validation cohort by the median value of each variable and recalculated the AUC of the model within each subgroup. As shown in Supplementary Table 2, the AUC remained high across all subgroups, ranging from 0.894 to 0.984. For SIRI, the low SIRI group achieved an AUC of 0.984 (95% CI: 0.940–1.000) and the high SIRI group achieved an AUC of 0.894 (95% CI: 0.762–1.000), with overlapping confidence intervals indicating no statistically significant difference. Similarly, for PASP, NLR, and NHR, the AUCs in the low and high subgroups were consistently above 0.92, and all confidence intervals overlapped substantially.

### 3.6 Calibration curve analysis

**Figure 6:**
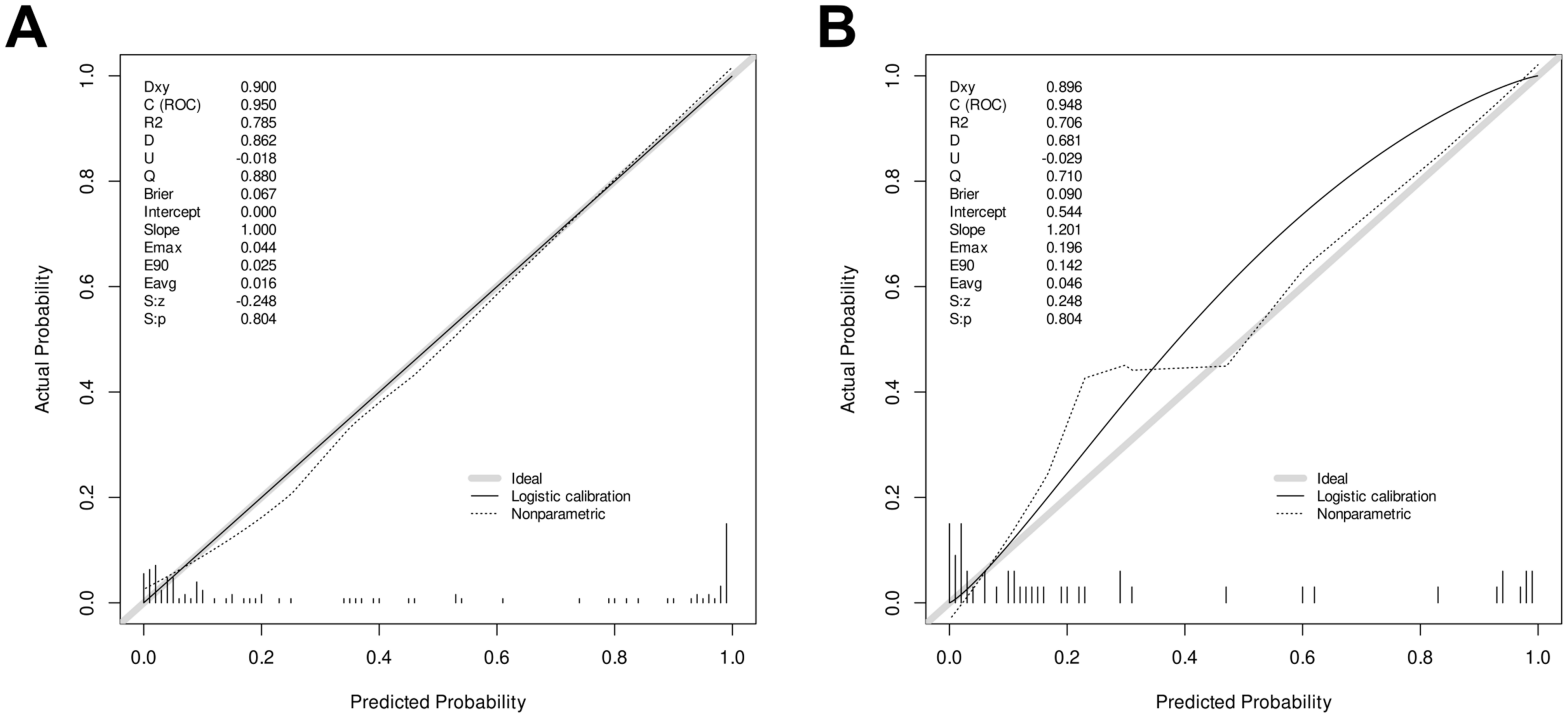
Calibration curve of the readmission prediction model for HHD-HF patients. **(A)** Training set. **(B)** Validation set.

The calibration plots of the nomogram in the different cohorts are plotted in the Figure 6, which demonstrate a good correlation between the observed and predicted Group. The results showed that the original nomogram was still valid for use in the validation sets, and the calibration curve of this model was relatively close to the ideal curve, which indicates that the predicted results were consistent with the actual findings.

Internal validation with 1,000 bootstrap resamples yielded a corrected C-index of 0.923 (95% CI: 0.845–0.981), confirming excellent discriminative performance and robustness of the nomogram without significant overfitting.

The Hosmer-Lemeshow goodness-of-fit test was performed to evaluate the calibration of the prediction model. In the training set, the test yielded a chi-square value of 6.20 (df = 8, *p* = 0.625), and in the validation set, the chi-square value was 3.17 (df = 8, *p* = 0.923), indicating no significant lack of fit and suggesting good calibration of the model.

### 3.7 Decision curve analysis

**Figure 7:**
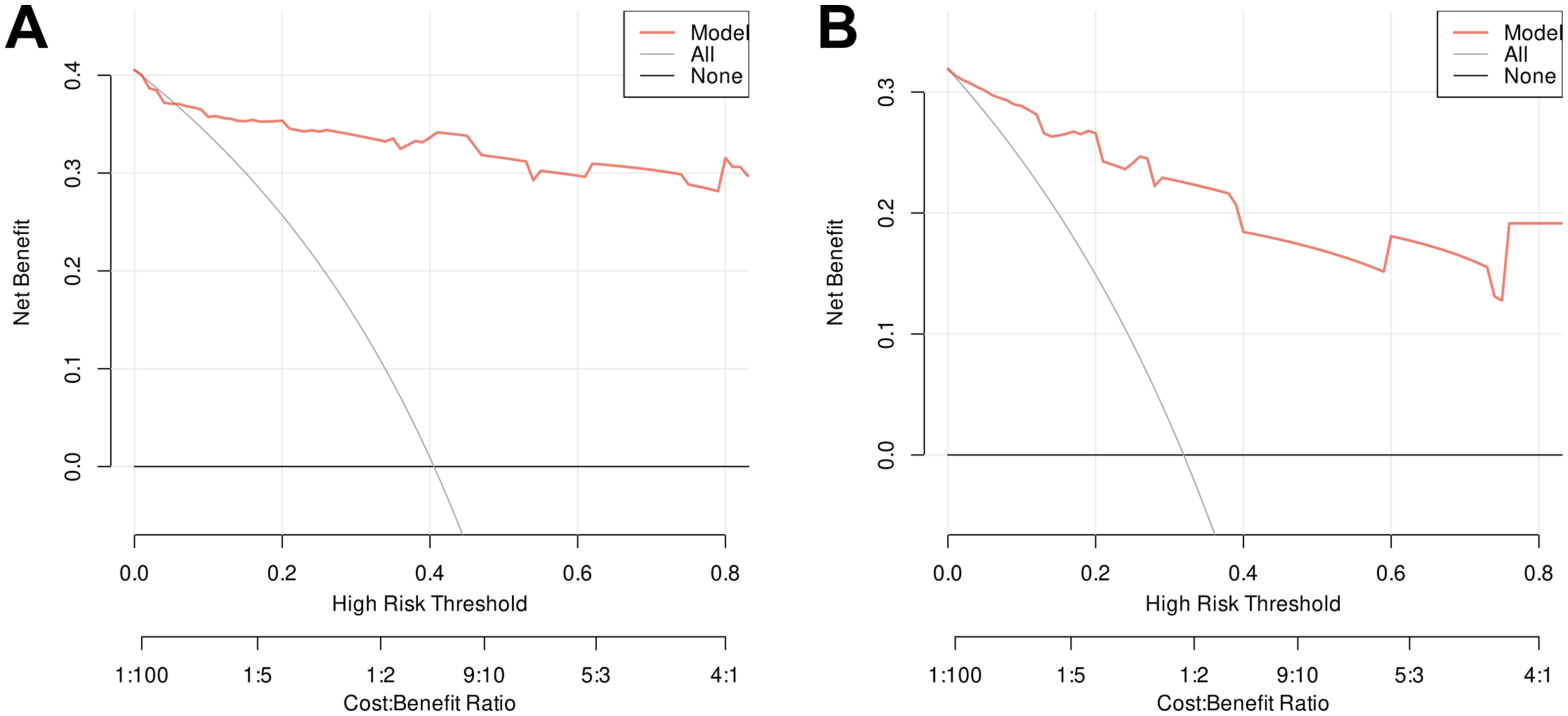
DCA curve of the predictive model for readmission in HHD-HF patients. **(A)** Training set. **(B)** Validation set.

The Figure 7 displays the DCA curves related to the nomogram. This research shows that the nomogram offers substantial net benefits for clinical application through its DCA curve. In training cohort (Figure 7A), the nomogram demonstrates positive net benefit when the risk threshold probability ranges from 0% to approximately 80%, with the highest net benefit observed at lower thresholds (5–20%). The nomogram (red line) consistently outperforms both the “treat all” strategy (grey line) and the “treat none” strategy (black line) across clinically relevant threshold ranges, indicating that using this prediction model to guide clinical decision-making would yield better outcomes than either treating all patients or treating no patients. Validation cohort (Figure 7B) shows comparable results, with the nomogram maintaining positive net benefit across threshold probabilities of 0–75% and similarly achieving optimal net benefit at thresholds of 5–20%.

### 3.8 Results of SHAP interpretability analysis

**Figure 8:**
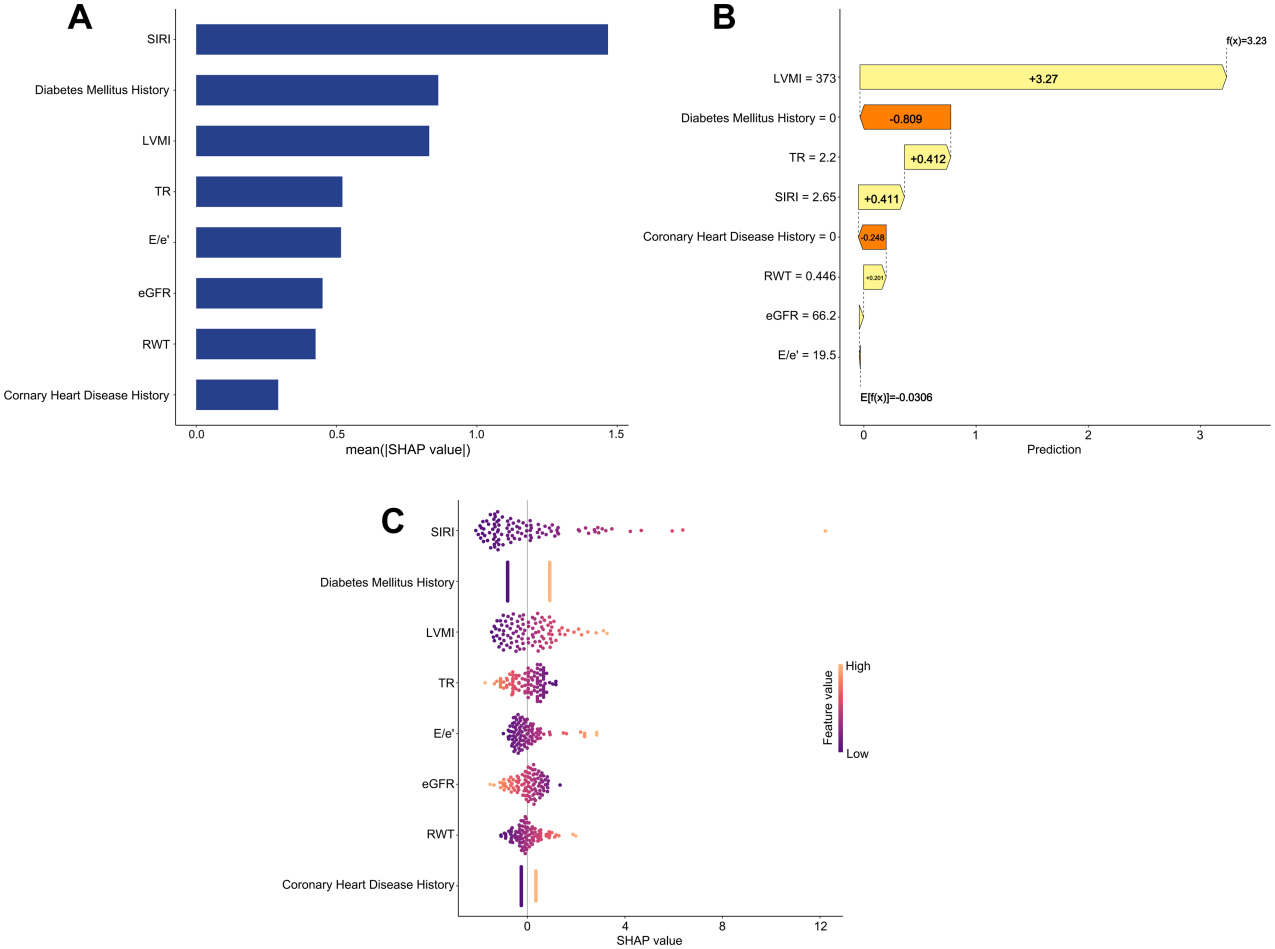
Model interpretability analysis based on SHAP. **(A)** Feature importance bar plot. This displays the ranking of variables based on their mean absolute SHAP values (mean(|SHAP value|)). A larger value indicates a greater overall contribution of that feature to the model's predictive outcome. **(B)** Single-sample SHAP waterfall plot/force plot. This illustrates how the prediction for a representative individual (f(x) = −3.23) is decomposed relative to the baseline expected value (E[f(x)] = 0.0306). Each bar represents the contribution of a feature: orange bars indicate that the feature pushes the prediction toward the negative direction (reducing risk/probability), while yellow bars indicate a push toward the positive direction (increasing risk/probability). The labeled values correspond to the SHAP values of each feature. **(C)** SHAP beeswarm plot/summary dot plot. This shows the distribution of SHAP values across all samples for each feature. The *X*-axis represents the SHAP value (negative values indicate a decrease in the predicted outcome, positive values indicate an increase); the *Y*-axis lists features sorted by importance. The color gradient (from purple to yellow/orange) reflects the variation in feature values from low to high, illustrating the relationship between feature magnitude and SHAP value. The *X*-axis represents the SHAP value (negative values indicate a decrease in readmission risk, positive values indicate an increase). The *Y*-axis lists features in descending order of importance. The color gradient from purple to yellow represents the feature value from low to high. For SIRI, LVMI, and diabetes history, higher feature values (yellower color) correspond to SHAP values shifted to the right, indicating an association with higher risk. For eGFR, higher feature values (yellower color) shift SHAP values to the left, indicating that higher eGFR is a protective factor.

To further elucidate the predictive mechanism of the nomogram and identify key contributing factors, this study employed the SHAP (SHapley Additive exPlanations) method for post-hoc model interpretation (Figure 8). Feature importance analysis (Figure 8A) revealed that the SIRI contributed the most to the model output (mean |SHAP| ≈ 1.4), far exceeding other clinical indicators; followed by diabetes mellitus history and LVMI, indicating that inflammatory status, metabolic derangement, and cardiac structural remodeling are the core factors for predicting the outcome. Figure 7B illustrates the prediction decomposition for a representative high-risk individual (baseline expected value E[f(x)] = −0.0306, individual prediction value f(x) = 3.23). This patient had a markedly elevated LVMI (373 g/m^2^, SHAP = +3.27), which was the primary driver increasing the predicted risk; although the absence of diabetes history (contribution value −0.809) exerted a protective effect, it could not offset the strong positive influence of LVMI, resulting in a high-risk category output (positive predictive value) by the model. The global feature-impact patterns are shown in Figure 8C. The beeswarm plot displays the direction of association between each variable and predicted risk: SIRI, LVMI, and diabetes history exhibited a clear positive-driving pattern—higher feature values (color shifting from purple to yellow) corresponded to SHAP values located further to the right (positive region), suggesting that elevated SIRI, increased LVMI, and the presence of diabetes are significantly associated with higher risk of adverse outcome. Similar trends were observed for TR and RWT. In contrast, the absence of diabetes history (low feature value, dark purple) predominantly clustered in the negative SHAP region (left side), reflecting its protective effect. Collectively, these results demonstrate that the model prioritizes inflammatory burden and myocardial remodeling indicators when making predictions, and the direction of each feature’s impact aligns with established clinical pathophysiological mechanisms.

### 3.9 Predictive performance of SIRI alone

**Figure 9:**
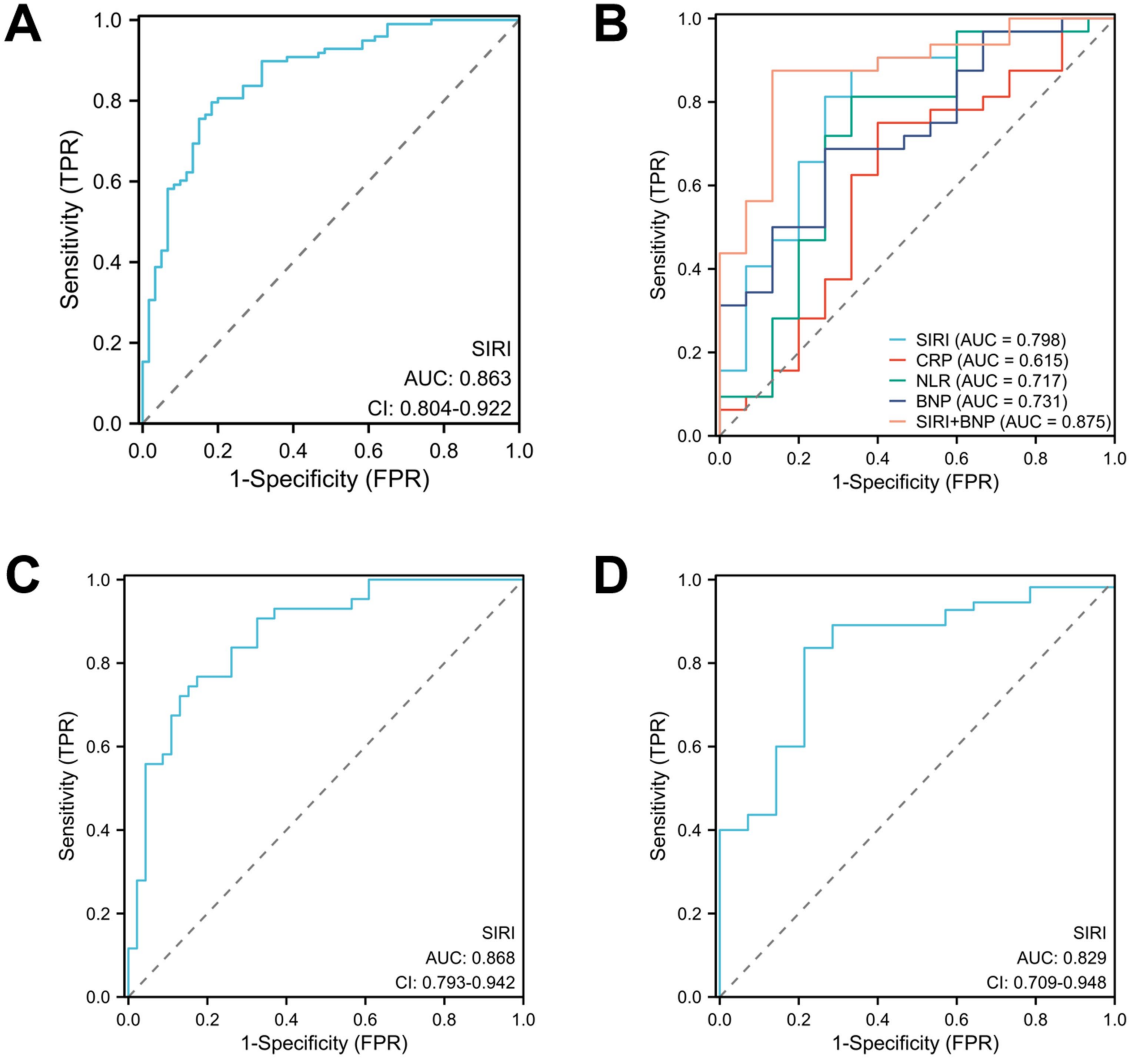
ROC curves of SIRI for predicting 6‑month readmission in different cohorts and comparison with other markers. **(A)** ROC curve of SIRI alone in the total cohort (*n* = 158). **(B)** Comparison of ROC curves for SIRI, CRP, NLR, BNP, and the combination of SIRI and BNP in the training cohort (*n* = 111). **(C)** ROC curve of SIRI in the HFrEF subgroup (LVEF < 40%, *n* = 89). **(D)** ROC curve of SIRI in the HFpEF subgroup (LVEF ≥ 40%, *n* = 69).

In the total cohort of 158 HHD-HF patients, the SIRI alone demonstrated good discriminative ability for 6-month readmission, with AUC of 0.863 (95% CI: 0.804–0.922). Using the Youden index, the optimal cut-off value of SIRI was determined as 1.71, with a sensitivity of 79.6% and a specificity of 81.7% (Figure 9A).

### 3.10 Comparison of predictive performance among inflammatory markers and BNP in the training cohort

In the training cohort (*n* = 111), we compared the discriminative ability of SIRI, CRP, NLR, BNP, and the combination of SIRI and BNP for predicting 6-month readmission. As shown in Table 5, SIRI alone achieved AUC of 0.884 (95% CI: 0.823–0.944), which was significantly higher than that of CRP (AUC = 0.710, 95% CI: 0.614–0.806; DeLong test *p* = 0.001), NLR (AUC = 0.818, 95% CI: 0.738–0.897; *p* < 0.001), and BNP (AUC = 0.693, 95% CI: 0.592–0.794; *p* < 0.001) (Figure 9B). The combined model of SIRI and BNP yielded an AUC of 0.882 (95% CI: 0.821–0.943), which was numerically similar to SIRI alone, and the DeLong test showed no statistically significant difference between SIRI alone and the combined model (*p* = 0.6848). These results indicate that SIRI outperforms conventional inflammatory markers (CRP and NLR) as well as BNP in predicting 6-month readmission, and adding BNP to SIRI does not provide incremental predictive value.

**Table 5:** Comparison of predictive performance among inflammatory markers and BNP in the training cohort.

Variable	*Z*	*P*
SIRI vs. CRP	3.314	0.001
SIRI vs. NLR	3.723	<0.001
SIRI vs. BNP	3.440	<0.001
SIRI vs. SIRI+BNP	0.406	0.685
CRP vs. NLR	−1.993	0.046
CRP vs. BNP	0.268	0.788
CRP vs. SIRI+BNP	−3.328	0.001
NLR vs. BNP	2.155	0.031
NLR vs. SIRI+BNP	−3.843	<0.001
BNP vs. SIRI+BNP	−3.513	<0.001

### 3.11 Subgroup analysis by heart failure phenotype

According to the left ventricular ejection fraction (LVEF), patients were classified into HFrEF (LVEF < 40%, *n* = 89) and HFpEF (LVEF ≥ 40%, *n* = 69) subgroups. The discriminative ability of SIRI for 6-month readmission was evaluated separately in each subgroup. In the HFrEF subgroup, the AUC of SIRI was 0.868 (95% CI: 0.793–0.942) (Figure 9C), while in the HFpEF subgroup, the AUC was 0.829 (95% CI: 0.709–0.948) (Figure 9D). The DeLong test showed no significant difference between the two AUCs (*Z* = 0.542, *p* = 0.589). Multivariate Logistic regression adjusting for the same covariates (RWT, E/e’, TR, LVMI, DM, CHD, eGFR) revealed that SIRI remained an independent predictor in both subgroups (HFrEF: OR = 3.650, 95% CI: 1.174–11.353, *p* = 0.025; HFpEF: OR = 3.330, 95% CI: 1.068–10.381, *p* = 0.038).

## 4 Discussion

In clinical practice, the readmission rate within 6 months after discharge for patients with HHD-HF is as high as 25 to 35%. This not only severely impairs patients’ quality of life and worsens prognosis, but also accounts for more than 70% of total heart failure healthcare expenditures, constituting a substantial burden (13). Inflammation is a key factor in the development and progression of HHD-HF. Myocardial remodeling and fibrosis induced by long-term hypertension are often accompanied by chronic inflammatory activation, and composite inflammatory indices obtainable from routine complete blood counts are closely associated with short-term adverse events in patients (14–16). However, there is currently a lack of targeted clinical assessment tools. Existing models primarily target the overall population with HF and fail to account for the unique pathophysiological characteristics of HHD-HF. Moreover, they struggle to integrate readily available inflammatory biomarkers for individualized risk stratification, leading to difficulties in early identification of high-risk individuals. This is a significant contributing factor to the persistently high readmission rates. Through retrospective cohort analysis, this study successfully developed and validated a clinical nomogram model for predicting the 6-month readmission risk in patients with HHD-HF. The model integrates three core categories of indicators: cardiac structure, metabolic comorbidities, and systemic inflammation, ultimately identifying LVMI, history of diabetes mellitus, and the SIRI as predictive factors. The SHAP explanatory analysis revealed that SIRI was the dominant variable driving model predictions (mean absolute SHAP value≈1.4), while correlation analysis showed significant positive associations between SIRI and rehospitalization risk (correlation coefficient R = 0.610) as well as LVMI (R = 0.413), a marker of left ventricular remodeling. Besides, the model demonstrated excellent discriminatory performance in both the training and validation sets, with AUC values of 0.950 and 0.948, respectively. These findings underscore the complex multifactorial nature of readmission risk in HHD-HF patients, driven by cardiac remodeling, metabolic disorders, and chronic inflammation. As a retrospective observational study, our findings can only confirm correlations, and the predictive value of SIRI requires validation through prospective studies.

Previous studies have demonstrated the correlation between left ventricular hypertrophy (LVH) and adverse cardiovascular outcomes, suggesting that LVH, as the core pathological change in HHD, may serve as a key predictor of readmission risk in HF patients (17–19). The findings of this study confirm this expectation and further highlight the advantages of LVMI over other echocardiographic parameters. From a mechanistic perspective, an increase in LVMI is a core marker of maladaptive cardiac remodeling caused by chronic pressure overload (resulting from long-term uncontrolled hypertension). Its essence lies in the cumulative effects of cardiomyocyte hypertrophy, myocardial fibrosis, and collagen deposition. This remodeling directly leads to diastolic dysfunction, further increases myocardial stiffness, and ultimately results in decompensation of myocardial contractile function and pump failure (20, 21). This study found that for every one-unit increase in LVMI, the probability of readmission increases by 1.02 times. This result is consistent with other literature reporting a positive correlation between the severity of LVH and adverse cardiovascular outcomes (22–24), further validating the value of LVMI. LVMI not only reflects the severity of cardiac structural remodeling but also indirectly indicates the duration and severity of uncontrolled hypertension, as well as the heart’s susceptibility to compensatory dysfunction under pressure load. Notably, this study found that other echocardiographic parameters, such as RWT and the E/e’ ratio, did not show independent significance in the multivariable model. We initially hypothesized that RWT (reflecting the geometric pattern of hypertrophy) and the E/e’ ratio (reflecting diastolic filling pressure) might synergistically predict risk alongside LVMI. However, the actual results indicated that in this specific population with established HHD-HF, LVMI has greater predictive value than the geometric pattern of hypertrophy or a single diastolic filling pressure indicator. The underlying mechanism may be that LVMI comprehensively encompasses all features of myocardial remodeling (including the overall extent of myocardial hypertrophy and fibrosis), whereas RWT only reflects the type of hypertrophy (concentric/eccentric) and may be associated with confounding factors such as inflammation (25). The E/e’ ratio primarily reflects the diastolic functional state and filling pressure at a single time point. Both of the latter fail to capture the complex and persistent cardiac remodeling state in HHD-HF patients as comprehensively as LVMI does. This finding provides a reference for subsequent research to prioritize LVMI as a core structural indicator.

The history of DM further highlighting the critical role of metabolic comorbidities in patients with HHD-HF. From a mechanistic perspective, the pathways through which DM exacerbates adverse outcomes in HHD-HF patients are multifaceted and align closely with the mechanism of metabolic dysregulation-myocardial injury-inflammatory activation. Firstly, DM directly aggravates myocardial injury; studies have shown that patients with type 2 DM and comorbid hypertension experience more severe impairment of left ventricular systolic function (26). Secondly, the core mechanism lies in insulin resistance, which has been causally linked to adverse left ventricular structural alterations (27). Simultaneously, DM often acts as a component of metabolic syndrome, triggering the activation of the ‘gut-heart axis’ from gut dysbiosis to systemic and local cardiac inflammation. This vicious cycle directly leads to cardiomyocyte hypertrophy, apoptosis, and fibrosis (28). This metabolic-inflammatory interplay also explains why the cumulative burden of metabolic syndrome components is associated with a dose-dependent surge in heart failure risk. Ultimately, these processes collectively result in significant cardiac remodeling, manifested as increased LVMI and RWT (29), thereby substantially elevating the risk of readmission. This finding from our study is consistent with broader research confirming the significant impact of cardiometabolic comorbidities on healthcare burden and mortality risk (30, 31). In particular, a study by Polat et al. noted that while acute events could act as a catalyst for improving guideline adherence in HF patients with comorbid coronary artery disease (CAD) and diabetes, this population still faces an extremely high risk of readmission (32). This finding aligns with and reinforces the results of the present study, further underscoring the persistent nature of DM as a high-risk factor. Our study positions DM as the foremost consideration in risk stratification for HHD-HF patients, clarifying that aggressive glycemic control and comprehensive metabolic health management are key interventional targets for reducing readmission rates in this population. This also provides an important basis for formulating subsequent clinical intervention strategies.

The inclusion of the SIRI as the third and highly significant predictor (OR = 2.88) in our model underscores the critical, independent role of low-grade persistent inflammation in driving adverse outcomes in HHD-HF. Its strong predictive power, further evidenced by SHAP analysis identifying it as the top contributor to the model’s output, highlights its central importance. From a mechanistic perspective, inflammation has been recognized as a core driver of all HF phenotypes (6). The advantage of SIRI lies in its ability to more finely reflect the activation state of the immune system: elevated neutrophils indicate enhanced inflammatory activation and oxidative stress, increased monocytes participate in the regulation of myocardial fibrosis and inflammatory responses, and decreased lymphocytes reflect immunosuppression. The integration of these three components provides a more comprehensive representation of the degree of immune dysregulation, whereas individual leukocyte subsets or traditional markers like CRP only reflect a single aspect of the inflammatory response (8). Our study found that CRP did not show significant predictive value in the final model. Compared to SIRI, CRP, as an acute-phase reactant protein, is more susceptible to the influence of short-term factors such as infection and stress, making it unable to stably reflect the long-term, low-grade persistent inflammatory state present in patients with HHD-HF. In contrast, SIRI can more reliably capture this chronic inflammatory process. Mechanistically, an elevated SIRI indicates a state of immune dysregulation and neutrophil-mediated oxidative stress, which continuously exacerbates myocardial injury, promotes myocardial fibrosis, and concurrently increases the risk of HF decompensation due to concurrent infections or aggravated inflammation (33). This finding aligns with emerging evidence in the field of cardiovascular diseases. For instance, in acute pulmonary embolism, inflammatory biomarkers such as the neutrophil-to-lymphocyte ratio are key components of prognostic nomograms (34). In cardiac surgery patients, integrating markers of systemic response can improve risk stratification for postoperative complications (35). By extending this paradigm to the chronic HHD-HF population, our study not only achieved its research objectives but also confirmed the advantages of SIRI as an “easily accessible, cost-effective, and prognostically significant” biomarker, providing a feasible indicator choice for risk assessment in primary healthcare institutions.

The nomogram developed in this study demonstrated excellent performance, with an AUC value exceeding 0.94. Its strength lies in the holistic integration of three distinct domains: specific cardiac structure (LVMI), key metabolic comorbidity (DM), and dynamic systemic processes (SIRI-related inflammatory response). In clinical practice, for patients presenting with concurrently high LVMI, uncontrolled diabetes, and elevated SIRI, physicians can intensify follow-up protocols, optimize guideline-directed therapy focusing on anti-remodeling and anti-inflammatory effects, enhance patient education, and facilitate close collaboration with a multidisciplinary team including dietitians and diabetes educators. The visual design of this nomogram aids in conveying risk information to patients, potentially improving adherence to treatment regimens. Furthermore, in resource-limited healthcare settings, such a tool can help prioritize the implementation of advanced management strategies and remote health monitoring for patients in greatest need. This aligns with the growing emphasis on personalized medicine and precision health in the management of chronic diseases.

This study found a significant positive correlation between SIRI and LVMI (R = 0.413, *p* < 0.001) This association is consistent with the findings reported by Hu et al. (36)in patients with atrial fibrillation. From a pathophysiological perspective, chronic pressure overload is the initiating factor for myocardial remodeling in hypertensive heart disease, directly inducing myocardial hypertrophy and fibrosis, accompanied by a sustained inflammatory response (37). As a composite indicator that comprehensively reflects the status of neutrophils, monocytes, and lymphocytes, an elevated SIRI marks the activation of core immune processes that drive myocardial fibrosis (38). Therefore, the results of this study support the theoretical model in which chronic inflammation plays a key driving role in the process of myocardial hypertrophy and fibrosis induced by stress overload. Furthermore, the significant negative correlation between SIRI and eGFR (R = -0.446, *p* < 0.001) aligns with the report by Wei et al. (39) regarding SIRI’s role in impairing renal function in a hypertensive population. The aforementioned associations support the role of the cardio-renal-inflammatory axis in HHD-HF (40). On one hand, chronic pressure overload and inflammation jointly drive myocardial hypertrophy and fibrosis; on the other hand, the systemic inflammatory state can also impair renal function directly or through pathways such as promoting atherosclerosis and endothelial dysfunction. These two processes form a vicious cycle that increases the risk of readmission (39). This study found that SIRI was significantly correlated with LVMI (*R* = 0.413) and eGFR (*R* = -0.446), delineating a potential vicious pathway: systemic inflammation activation may promote pressure overload-induced myocardial hypertrophy and fibrosis, while simultaneously exacerbating renal impairment. These three factors form a vicious cycle that collectively elevates the patient’s risk of readmission. Conversely, SIRI showed no significant correlation with the severity of tricuspid regurgitation, suggesting that SIRI reflects a systemic pathophysiological state rather than a simple right heart hemodynamic abnormality.

Notably, the predictors that did not maintain independent significance in the final model of this study history of CHD, eGFR, and BNP hold implications. Given the prevalence of ischemic etiology in HF, the non-significance of CHD history may seem counterintuitive. However, in studies focusing specifically on the HHD population, the primary pathophysiological mechanism is pressure overload rather than epicardial coronary artery disease (41). Although ischemia may be concomitant, its contribution to short-term readmission risk in this specific population might be overshadowed by the dominant effects of hypertrophy, metabolic disease, and inflammation. We further speculate that in the HHD-HF subgroup, where pressure overload is the dominant pathophysiological driver, the prognostic impact of coronary artery disease as a concomitant factor may be masked or mediated by the more core axis of myocardial remodeling (LVMI), metabolic dysregulation (DM), and systemic inflammation (SIRI). The chronic inflammatory state reflected by elevated SIRI may partially capture the pro-inflammatory effects of coronary atherosclerosis, while LVMI and DM already integrate the consequences of pressure load and metabolic stress, thereby attenuating the independent predictive value of CHD history in the multivariable model. The lack of independence for eGFR and BNP is particularly noteworthy. Renal dysfunction and elevated natriuretic peptides are well-established prognostic markers in the general HF population (42). Their exclusion from the final model suggests that, within the HHD-HF context, the information these markers provide regarding hemodynamic stress and renal function may be partially captured or replaced by the triad of LVMI, DM, and SIRI. This does not diminish their overall clinical importance but underscores that the relative weight of variables can change in predictive modeling for specific subpopulations.

In further exploratory analyses, we evaluated the predictive performance of SIRI alone and compared it with other inflammatory markers and BNP, as well as across heart failure phenotypes. SIRI alone achieved AUC of 0.863 (95% CI: 0.804–0.922) for predicting 6-month readmission in the total cohort, with an optimal cut-off value of 1.71 (sensitivity 79.6%, specificity 81.7%). When compared with conventional inflammatory markers and BNP in the training cohort, SIRI significantly outperformed CRP (AUC 0.884 vs. 0.710, *p* = 0.001), NLR (AUC 0.884 vs. 0.818, *p* < 0.001), and BNP (AUC 0.884 vs. 0.693, *p* < 0.001). Notably, adding BNP to SIRI did not improve predictive performance (AUC 0.882, *p* = 0.685 vs. SIRI alone), indicating that SIRI alone captures the prognostic information provided by BNP in this specific population. These findings suggest that, in our study, SIRI demonstrated superior predictive performance compared to traditional single inflammatory markers, and clinically, SIRI can serve as an auxiliary predictive biomarker for 6-month readmission in patients with HDD-HF. However, these results should be interpreted as hypothesis-generating, and further external validation in larger, multicenter cohorts is required before clinical implementation.

Furthermore, subgroup analysis based on LVEF showed that SIRI maintained good discriminative ability in both HFrEF (AUC = 0.868, 95% CI: 0.793–0.942) and HFpEF (AUC = 0.829, 95% CI: 0.709–0.948), with no significant difference between the two AUCs (*p* = 0.589). Multivariable logistic regression adjusting for the same covariates confirmed that SIRI remained an independent predictor in both HFrEF (OR 3.65, 95% CI: 1.17–11.35, *p* = 0.025) and HFpEF (OR 3.33, 95% CI: 1.07–10.38, *p* = 0.038). These results demonstrate that the prognostic value of SIRI is consistent across the spectrum of heart failure phenotypes in HHD-HF patients, further supporting its utility as a robust and versatile biomarker.

However, this study also has certain limitations. First, as a single-center retrospective study with a limited sample size (*n* = 158) and without external validation, the findings require confirmation in larger, multicenter, prospective cohorts. Second, we lacked longitudinal SIRI measurements and detailed discharge medication records (including SGLT2 inhibitors and ARNI), which may affect the predictive performance of SIRI. Third, the primary analysis employed a binary logistic regression model that did not account for the competing risk of mortality. Fourth, despite the use of telephone follow-up and EMR review from four collaborating institutions, single-center data may have missed readmissions occurring at external hospitals. Finally, due to incomplete documentation in our retrospective database, we were unable to perform subgroup analyses stratified by NYHA class or diabetes severity, which may limit the precision of SIRI application across different risk-stratified populations. Despite these limitations, this study provides preliminary evidence for the potential value of SIRI in predicting 6-month readmission risk at discharge in patients with HHD-HF.

## 5 Conclusion

In summary, this study successfully developed and validated a clinical nomogram integrating LVMI, diabetes history, and SIRI to predict the 6-month readmission risk in patients with HHD-HF. The nomogram demonstrated excellent discriminative ability (AUC > 0.94 in both training and validation cohorts), good calibration, and net clinical benefit. When used alone, SIRI achieved an AUC of 0.863 (optimal cut-off 1.71), with predictive performance significantly superior to CRP, NLR, and BNP. Subgroup analyses confirmed that SIRI maintained stable predictive performance in both HFrEF and HFpEF phenotypes. SHAP analysis highlighted SIRI as the dominant contributor, emphasizing the role of chronic inflammation in HHD-HF progression. The model enables individualized risk assessment, early identification of high-risk patients, and guided follow-up. However, as a retrospective observational study, these findings primarily reveal correlations and the preliminary predictive potential of the model. The definitive clinical value of SIRI requires validation through prospective multicenter studies. Future research should also explore whether dynamic monitoring of SIRI provides additional information for assessing treatment response.

## Data Availability

The raw data supporting the conclusions of this article will be made available by the authors, without undue reservation.

## Footnotes

1
www.mstata.com
