# Supplementary material for: The correlation between the systemic inflammation response index and 6-month readmission risk in patients with hypertensive heart disease-related heart failure and its predictive model
Source: Front Med (Lausanne). 2026 May 28;13:1799853. doi: 10.3389/fmed.2026.1799853 (PMC13253545; doi:10.3389/fmed.2026.1799853)
Supplement: Supplementary file 1 [file Table_1.docx]

**Supplementary Table 1** Collinearity analysis results

| Item | VIF | Tolerance |
| --- | --- | --- |
| Diabetes Mellitus History | 1.301 | 0.769 |
| Coronary Heart Disease History | 1.330 | 0.752 |
| RWT | 1.420 | 0.704 |
| E/e’ | 1.530 | 0.654 |
| TR | 1.256 | 0.796 |
| LVMI | 1.504 | 0.665 |
| SIRI | 1.437 | 0.696 |
| eGFR | 1.430 | 0.699 |

**Supplementary Table 2 Subgroup analysis of nomogram performance in the validation set**

| Variable | Subgroup | N | AUC | 95% CI |
| --- | --- | --- | --- | --- |
| PASP (mmHg) | Low (≤ 27.0) | 25 | 0.924 | 0.764 ~ 1.000 |
|  | High (> 27.0) | 22 | 0.950 | 0.860 ~ 1.000 |
| NLR | Low (≤ 1.99) | 24 | 0.950 | 0.860 ~ 1.000 |
|  | High (> 1.99) | 23 | 0.947 | 0.865 ~ 1.000 |
| SIRI | Low (≤ 1.12) | 24 | 0.984 | 0.940 ~ 1.000 |
|  | High (> 1.12) | 23 | 0.894 | 0.762 ~ 1.000 |
| NHR | Low (≤ 4.19) | 24 | 0.953 | 0.876 ~ 1.000 |
|  | High (> 4.19) | 23 | 0.973 | 0.914 ~ 1.000 |

Note: Subgroups were defined using the median value of each variable within the validation set.
